# Appraisal of Saliva and Its Sensory Perception in Reproductive Transitions of Women: A Review

**DOI:** 10.7759/cureus.31614

**Published:** 2022-11-17

**Authors:** Anshu T Agrawal, Alka Hande, Amit Reche, Priyanka Paul

**Affiliations:** 1 Intern, Sharad Pawar Dental College, Datta Meghe Institute of Medical Sciences (Deemed to be University), Wardha, IND; 2 Department of Oral Pathology and Microbiology, Sharad Pawar Dental College, Datta Meghe Institute of Medical Sciences (Deemed to be University), Wardha, IND; 3 Department of Public Health Dentistry, Sharad Pawar Dental College, Datta Meghe Institute of Medical Sciences (Deemed to be University), Wardha, IND

**Keywords:** ph, salivary stream rate, menopause, pregnancy, menstruation

## Abstract

There are physical, hormonal, and psychological variations in women, which affect their health in general. This may influence the environment of oral cavity, specific to salivary stream rate, salivary pH, and buffering capacity. Saliva is a perplexing liquid containing an assortment of mucosal host protection factors from diverse salivary organs and crevicular liquid. Though saliva has been inspected with regard to several physiological and pathological conditions, the association of various properties of saliva with different phases of women's menstrual cycle remains unexplored. Because diet and salivary stream rate are correlated, food not only affects salivary flow but also has an impact on sensory perception. One of the most vital human senses, taste, is crucial in determining a person's dietary requirements, which in turn influences eating habits and, eventually, human nourishment. As a result, along with variations in salivary stream rate and pH, the capability to sense taste may also alter during the phases of pregnancy, menstruation, and menopause. Hence, this review article is designed to assess salivary stream rate, pH, and gustatory function in several phases of women's lives to determine the impact of menstruation, pregnancy, and menopause on saliva as well as the reciprocal relationship between saliva and gustatory function.

## Introduction and background

Women's health requirements differ primarily as a result of the unique changes that take place during their lifetimes. This is because hormonal variances happen throughout a woman's life, influencing the physiology of the whole body involving the oral cavity. As a result, a variety of phases such as pregnancy, menstruation, and menopause may either have a direct impact on the metabolism of periodontal tissues or could prompt alterations in salivary stream rates, buffering limit, and taste discernment [1,2].

Saliva is the most readily available biofluid of human metabolism, flushing the oral cavity tenaciously and working to control its environment. It is believed to be a proactive gateway for the body to forecast well-being and illness. The examination of saliva and its numerous inorganic and organic components aid in the monitoring of physiologic and pathologic mechanisms [3]. The role of saliva consists of lubrication, mastication, taste discernment, and reduction of tooth cavities and oral infections [4]. Both quantitative and qualitative methods can be used to evaluate changes in salivary production. Assessment of salivary components may provide information about a person's overall health and is thought to reflect the body's well-being. Therefore, using saliva as a diagnostic fluid in sickness is an alluring justification.

In general, ovarian hormones significantly influence a woman's life. Throughout menstruation, there are alterations in estrogen and progesterone levels. For several women, the appearance of monthly menses brings out related oral changes [5]. There have been reports of oral inconveniences such as mild burning sensation, bleeding with mild irritation, gum redness, recurring oral ulcers, herpes labialis, candida albicans infection, increased tooth mobility, and emotional upsets [5-10]. Distinct menstrual cycle stages and varying hormone levels are linked to gingival diseases [11,12]. Hormonal disturbances are thought to cause gingival abnormalities in menstruation, which can occasionally be seen in conjunction with a propensity for ovarian dysfunction. Variations in steroidal sex hormone levels during menstruation may be due to modifications in the immune system [13].

Numerous research on humans suggests that hormonal changes during ovulation affect the content of saliva. Due to intricate hormonal connections, there are significant physiologic changes throughout pregnancy [14]. The health of soft tissues in the oral cavity may be jeopardized by alterations in salivary content and stream rate. Female hormonal fluctuations in the oral cavity during pregnancy may cause transient alterations in salivary stream rate, buffering limit, and biological content of saliva, in addition to its direct impact on the metabolism of periodontal tissue. Pregnancy raises the risk of pregnancy gingivitis, a kind of gingival inflammation with an increased propensity to bleed without a specific plaque connection. Pregnancy can also lead to an increment in periodontal pocket development and dental caries. These alterations are reversible upon delivery, albeit the precise cause is still unknown [15]. The content of saliva is crucial for reducing the likelihood of tooth decay [16].

Menopause causes specific hormonal alterations in women, making them more susceptible to salivary changes, such as increased sensitivity to hot and cold food, modified taste, burning feeling in the mouth, and decreased saliva flow that can cause xerostomia. Following menopause, the functions of taste buds and related neural network change due to decrease in the production of saliva, which results in dysesthesia and atrophic gingivitis [17]. As estrogen levels decline with menopause, there is higher incidence of bone density loss, particularly in the jaws, which can result in tooth loss. Hence, there can be a straightforward association between women's dental health and fluctuating hormone condition [18]. Although there are some variations that have been linked to menopause, the precise cause and the methods by which these signs and symptoms first appear are still undetermined [19].

Because diet and salivary stream rate are correlated, food not only affects salivary flow but also sensory perception. One of the most vital human senses, taste, is crucial in determining a person's dietary requirements, influencing eating habits and, eventually, human nourishment. As a result, along with variations in salivary stream rate and pH, the capability to sense taste may also alter during pregnancy, menstruation, and menopause [20].

Several widely employed electronic databases, including PubMed, Google Scholar, Web of Science, Scopus, Science Direct, and Medknow were utilized to search relevant information from the literature using keywords like "pregnancy," "menstruation," "menopause," "saliva," and "gustatory perception." Most research projects have concentrated on the importance of salivary stream rate, salivary pH, and sex-dependent variations in taste discernment in the establishment of caries. But salivary stream rate, pH, and taste discernment in menstrual period, pregnancy, and menopausal women have been rarely analyzed in a single research work. With this premise in mind, this review article is designed to assess salivary stream rate, pH, and gustatory perception in several phases of reproductive women to determine the impact of menstrual cycle, pregnancy, and menopause on saliva as well as the reciprocal relationship between saliva and gustatory function.

## Review

Several milestones in a woman's lifespan are influenced by hormonal alterations at various points in the reproductive cycle. Women are especially susceptible to oral health problems during these periods. Puberty, the monthly menstrual cycle, the stage of birth control pill use, the time of pregnancy, and menopause are few examples of these stages. The maintenance of oral health requires saliva. Without saliva, meals are difficult, uncomfortable, and unpleasant [21]. Women's health requirements change during the course of their lives primarily as a result of hormonal alterations. Therefore, menstruation cycle and menopause may disrupt the metabolism of periodontal tissue, resulting in enlarged salivary glands, bright red swollen gums, or bleeding gums, or may cause changes in salivary stream rate, pH, and taste perception [2].

Saliva and menstruation

Salivary Flow Rate

Regarding the salivary stream rate, menstruating women showed no statistically significant change. Saluja et al. conducted a study on 120 subjects with 30 controls (with normal reproductive cycle) and 90 cases (30 individuals with three days of menstrual cycle, 30 pregnant, and 30 post-menopausal) [22]. They collected paraffin-induced salivary specimens by expectoration to assess salivary stream rate, gustatory perception, and pH. Then, at that point, an entire mouth trial was acted, in which the quality recognizable proof and force evaluations of taste arrangements were estimated. No measurably critical distinction was found between the gatherings concerning salivary stream rate.

The stage of menstruation did not affect the paraffin-stimulated salivary stream rate, according to research by Laine et al. [23]. On the contrary, Kullander and Sonesson demonstrated that histamine-stimulated production was marginally higher during the luteal phase of menstruation [24].

Gustatory Perception

Neurochemical, hormonal, physiological, and psychological elements have an impact on food consumption and choice. The neurochemical modality of taste is crucial for preserving an excellent nutritional status [25]. It is observed that people's preferences for sucrose change as the menstrual cycle progresses along with alterations in eating habits and other gustatory preferences [26].

Numerous researchers have reported that women at the time of the luteal phase of their menstruation cycle particularly like salty foods. This is consistent with the observations made by Frye and Demolar, who also noted an enhancement in salt preference during the luteal phase [27].

According to a study by Barbosa et al., during the luteal phase of menstruation cycle, there is alteration in taste perception, specific to sour taste. This results in poor eating habits leading to disturbances in metabolism and illness like obesity [28]. It is also observed that during the period of ovulation and pre-menstruation, there is alteration in serum estradiol levels resulting in gingival bleeding. Apart from these, there are periodic gingival alterations due to hormonal imbalance and ovarian dysfunction during menstruation.

Salivary pH

Salivary buffering ability is mainly reliant on the salivary stream rate, resulting in low concentration of saliva in situations where the salivary flow is restricted. Studies have revealed that the levels of buffering capacity do not change throughout the menstrual period [24]. Especially in comparison to pregnancy, constancy of salivary buffering limits in menstruation can be because of restricted hormonal change that happens throughout a brief phase of menstruation.

Saliva and pregnancy

Salivary Flow Rate

Overall, studies have shown that the salivary stream rate is lower in pregnant women compared to non-pregnant women. Research work carried out by Karnik et al. and Hugoson revealed identical detection of reduced salivary stream rate in pregnant women [29,30]. Progesterone and estrogen (estradiol, estriol, and estrone) levels rises gradually and significantly at the time of pregnancy due to placental secretion of these hormones. The salivary stream rate decreases as progesterone and human chorionic somatomammotropin (HCS) level rises [14].

Gustatory Perception

Saluja et al. discovered that pregnant women had lower total intensity rating for sucrose, but the results were not statistically significant [22]. On the contrary, average overall taste intensity ratings for sodium chloride, citric acid, and quinine hydrochloride are insufficient to draw the conclusion that there is decline in sensitivity to salty, sour, and bitter tastes. In their study, Kuga et al. found that pregnant women preferred acidic/sour flavors [31]. Reduced gustatory sensitivity occurs during pregnancy, making it more difficult for the mother to ingest enough electrolytes and provide her unborn child with a balanced diet. 

Salivary pH

The pH of saliva is influenced by salivary stream rate. Hence, salivary pH is higher at higher flow rates. This may be due to rise in bicarbonate content with an increase in flow rate [16]. According to studies, pregnant women's salivary pH is lower than non-pregnant women's.

When Rosenthal et al. analyzed the pH of saliva in pregnant and non-pregnant women, they found that pregnant women's saliva had an average pH of 6.5 [32]. In contrast, non-pregnant women's saliva had an average pH of 7.0. In earlier studies, Kullander and Soneson and Hegde et al. noted lower salivary pH in pregnant women [24,33]. According to Laine et al., salivary pH falls as the pregnancy progresses and then quickly increases after delivery [34]. Rockenbach et al. reported that pregnant women have lower salivary pH than non-pregnant women [2]. Additionally, the elevated progesterone lowers the plasma bicarbonate level, which reduces the pH of saliva.

According to Percival et al., an inverse correlation exists between population's vulnerability to caries and saliva's ability to act as a buffer [35]. Russell et al. revealed that, theoretically, there is an indirect association between the secretory rate of oral fluid, Decayed, Missing, Filled Teeth (DMFT) index, and caries susceptibility [36]. Mandel and Russell et al. suggested that the relationship between salivary secretion rate and caries incidence was only marginal or nonexistent [21,36]. It was also found that nutritional changes, vomiting, variations in oral microbiota and oral fluid, dietary changes, and poor maintenance of the oral cavity have a vital role in the development of caries during pregnancy. On the other hand, Karnik et al. asserted that there is a direct relation between pregnancy and dental caries [29].

Saliva and menopause

Oral discomforts, including burning feelings, have long been known to be closely related to menopause. Several distinctive changes come with menopause, some of which affect the mouth. It has been demonstrated that estrogen can impact a variety of oral tissues, including temporomandibular joints, oral mucosa, salivary glands, and jaw bones. Following menopause, variations in brain networks and taste buds functionality has been observed [37].

Salivary Flow Rate

It has been noted that from the start of menopause until full menopause, the salivary flow rate gradually declines. The difference, however, was not shown to be statistically noteworthy. These outcomes are consistent with Rokenbach et al., even though Palomares et al. and Harirah et al. have shown no change in the stream rate of saliva after menopause [2,38,39].

It is also found that geriatric aging can give rise to decline in the salivary stream rate due to decreased parenchymal tissue [40]. Patients with poor salivary flow rates have increased chances of dental cavities, dysphagia, oral mucositis, and changing taste. Hence, they should take an active role in maintenance of oral cavity by lubricating their mouths with artificial saliva or salivary substitute to replace lost moisture.

Gustatory Perception

Nutrition is essential during menopause as nutritional status can impact chemosensory function and impairment resulting in dietary changes [17]. Saliva and diet are interdependent. Therefore, dietary behavior and nutritional status can affect salivary production and sensory perception in addition to salivary flow. As a result, menstruation and menopause may potentially modify the capability for taste perception along with changes in salivary flow rate and pH [20].

Various studies concluded that post-menopausal women have decreased sweet intensity and decreased mean total intensity of fructose. These outcomes are in accordance with Delilbasi et al. and Saluja et al. [17,22]. A decreased sucrose sensation in post-menopausal women can be connected with an adjusted diet. Alterations in different receptors, including taste organ, can be brought about by aging. As there is no direct correlation between menstruation, menopause, and taste alteration, there can be an increased incidence of oral discomfort among women due to hormonal fluctuations, including complaints of candidial infections, burning sensation in the oral cavity, swollen salivary glands, and mood alteration. Wardrop et al. observed an increased frequency of “oral discomfort” in menopausal as well as post-menopausal women [41].

Salivary pH

Concerning pH, it has been observed that post-menopausal women have significantly decreased salivary pH than menstruating and pregnant women, which can lead to higher DMFT and Oral Hygiene Index-Simplified (OHI-S) score in both menstruating and pregnant women. Also, higher incidences of dental caries and oral infections can be noted in individuals with lower pH. According to Mahesh et al. and Rukmini et al., salivary pH is less in menopausal women when compared to menstruating and pregnant women [1,42]. In women, salivary pH is highly correlated with plasma adiponectin levels, which are a marker of pro-inflammatory cardiometabolic risk tolerance related to type II diabetes and obesity. Salivary pH is determined by various components of cardiometabolic risk like oxidation, inflammation, and many health modulators consisting status of menopause. It follows that salivary pH could be a possible measure for cardiometabolic risk profile analysis [43].

**Table 1:** Phases of reproductive transitions of women and its corelation with saliva and its parameters

Reproductive Transitions	Salivary Parameters		
	Salivary flow rate	Gustatory perception	Salivary pH
Menstruation	No significant change	Alterations in sweet, salty and sour taste	No significant change
Pregnancy	Decreased	Preferred acidic/sour flavors	Decreased
Menopause	Significantly decreased	Decreased sweet intensity	Significantly decreased

**Table 2:** Literature review

Author (Year)	Aim	Finding
Mahesh D.R.(2014) [1]	To measure and contrast the salivary stream rate, buffering limit, and pH of resting and stimulated saliva in pre, post, and post-menopausal women receiving hormone replacement therapy.	Salivary stream rate and pH were much reduced; while buffer limits were higher in post-menopausal women when compared to menstruating women. Also, people on HRT experienced an improvement in stream rate.
Cagri Delilbasi (2003) [17]	Aims to assess postmenopausal women's gustatory functioning.	In postmenopausal women, palate sensitivity and sucrose sensation significantly decreased with increase in tendency to consume sugary food.
Pulin Saluja (2014) [22]	To assess how menstruation, pregnancy, and menopause affect salivary stream rate, pH, and gustatory perception.	Decreased pH and flow rate of saliva in postmenopausal women. Additionally, pregnant and postmenopausal ladies showed decreased perception of sucrose, leading to the consumption of more sugary foods, while there is no discernible difference among the participants with respect to other flavors.
Amruta A. Karnik (2015) [29]	In order to contrast and associate the salivary stream rate, pH, and incidence of tooth decay in pregnant and non-pregnant ladies, the research examined salivary stream rate and pH in both groups.	When compared to non-pregnant ladies, pregnant ladies had reduced salivary stream rate, and their pH levels were likewise lower than those of non-pregnant ladies. Both group demonstrated a strong negative connection between pH and the DMFT score.
Shweta Hegde (2016) [33]	Sought to compare pregnant and non-pregnant women's salivary stream rate, pH, buffering capacity, calcium, and overall protein levels.	Pregnant women's salivary stream rate and pH levels are lower than in non-pregnant women. Both pregnant, and non-pregnant women's DMFT indexes demonstrated a high connection with pH respectively.
Usha Balan (2012) [44]	To examine the varied oral cavity signs and clinical characteristics in healthy women during the typical monthly cycle.	No participant noted change in flavor perception while aphthous ulcers were reported by 30% of research participants, herpes labialis by 5%, depression by 25%, and gingival bleeding by 8%.
Aleksandra Ciesielska (2022) [45]	To identify the most common oral variations in menopausal ladies, with a focus on the physicochemical and compositional alterations in saliva.	Salivary stream rates are influenced by an individual's estrogen status; menopausal women have relatively less salivation than women in the reproductive period.

## Conclusions

Hormonal variations happen throughout a woman's life, influencing the physiology of the whole body involving the oral cavity. This may prompt changes in salivary stream rates, buffering limit, and taste discernment or could have a direct impact on the metabolism of periodontal tissue. It has been observed that in context of the salivary flow rate and pH, there is no significant alteration seen during menstruation, but variation in sweet, salty, and sour taste is noted. In the phase of pregnancy, decrease in salivary flow rate and pH has been observed, with a preference for acidic/sour flavors. During menopause, there is significantly decreased salivary flow rate and pH as well as decreased sweet intensity.
